# Construction and Characterization of a Cellulolytic Consortium Enriched from the Hindgut of *Holotrichia parallela* Larvae

**DOI:** 10.3390/ijms17101646

**Published:** 2016-09-30

**Authors:** Ping Sheng, Jiangli Huang, Zhihong Zhang, Dongsheng Wang, Xiaojuan Tian, Jiannan Ding

**Affiliations:** Institute of Biological Resources, Jiangxi Academy of Sciences, Nanchang 330096, China; jiangli_35@tom.com (J.H.); zzh04scau@126.com (Z.Z.); w_d_sh@126.com (D.W.); Tianxiaojuan114@163.com (X.T.); jiannanding@aliyun.com (J.D.)

**Keywords:** cellulolytic consortium, enrichment, rice straw degradation, pyrosequencing, *Holotrichia parallela* larvae

## Abstract

Degradation of rice straw by cooperative microbial activities is at present the most attractive alternative to fuels and provides a basis for biomass conversion. The use of microbial consortia in the biodegradation of lignocelluloses could reduce problems such as incomplete synergistic enzymes, end-product inhibition, and so on. In this study, a cellulolytic microbial consortium was enriched from the hindgut of *Holotrichia parallela* larvae via continuous subcultivation (20 subcultures in total) under static conditions. The degradation ratio for rice straw was about 83.1% after three days of cultivation, indicating its strong cellulolytic activity. The diversity analysis results showed that the bacterial diversity and richness decreased during the consortium enrichment process, and the consortium enrichment process could lead to a significant enrichment of phyla Proteobacteria and Spirochaetes, classes Clostridia, Epsilonproteobacteria, and Betaproteobacteria, and genera *Arcobacter*, *Treponema*, *Comamonas*, and *Clostridium*. Some of these are well known as typical cellulolytic and hemicellulolytic microorganisms. Our results revealed that the microbial consortium identified herein is a potential candidate for use in the degradation of waste lignocellulosic biomass and further highlights the hindgut of the larvae as a reservoir of extensive and specific cellulolytic and hemicellulolytic microbes.

## 1. Introduction

Nowadays, an energy crisis and environmental pollution are global concerns. For the sustainable production of fuel, the development of renewable biological resources, which are considered economic and environmentally sound alternatives to finite fossil fuels is imperative [1]. Lignocellulosic biomass (such as that of rice straw, cotton straw, corn stover, etc.) is the most abundant and renewable source on Earth, and use of lignocellulosic biomass as a renewable source of energy and fuels is of great interest [2,3]. Among them, rice straw is one of the most abundant lignocellulosic waste materials in the world; thus, it is an attractive lignocellulosic material for the production of bioethanol. Because of the heterogeneous complex of carbohydrate polymers in rice straw, challenges related to pretreatment and enzymatic hydrolysis have prevented its widespread conversion to biofuel. Previous studies have shown that lignocellulosic materials (such as rice straw) are efficiently degraded through the cooperative activities of many microorganisms [3,4]; this has several advantages over monocultures (pure cultures), including better adaptation to changing conditions, enhanced substrate utilization, and higher cellulolytic activity [5].

The enrichment culture technique is a powerful tool for obtaining microbial consortia with desired cellulolytic properties [6], and the microbial source plays an important role in obtaining functional microorganisms when the enrichment conditions have been set [3]. In recent years, several cellulose-degrading consortia have been enriched from different ecosystems, such as soils [3,7], composts [4,8], etc. However, some phytophagous insects, such as termites, wood-feeding roaches, and beetles, have not yet received enough attention. Previous studies have shown that there are many cellulolytic and hemicellulolytic microorganisms in their gut, and they are considered to be efficient (hemi)cellulose degradation systems [9].

The experimental insect-phytophagous scarab larvae-live in the soil, where they feed on plant roots and organic matter of low nutritive value [10]. The hindgut of the larvae is enlarged and typically contains a wide diversity of microorganisms. From our previous studies, many cellulolytic and hemicellulolytic bacteria and enzymes have been isolated from the hindgut of *H. parallela* larvae [11,12,13]. These studies demonstrated that the scarab gut is a prospective resource for the isolation of many cellulolytic and hemicellulolytic microorganisms and enzymes, and they might be considered a potential source for bio-fuel production [14]. However, up to now, the cellulolytic and hemicellulolytic consortia isolated from the hindgut of phytophagous scarab have not yet received enough attention.

In the present study, a strong (hemi)cellulolytic microbial consortium was enriched from the hindgut of *H. parallela* larvae to degrade rice straw, and next-generation sequencing techniques were used to assess the stability and structure dynamics of this microbial community during the consortium enrichment process.

## 2. Results

### 2.1. Consortium Enrichment

**Figure 1:**
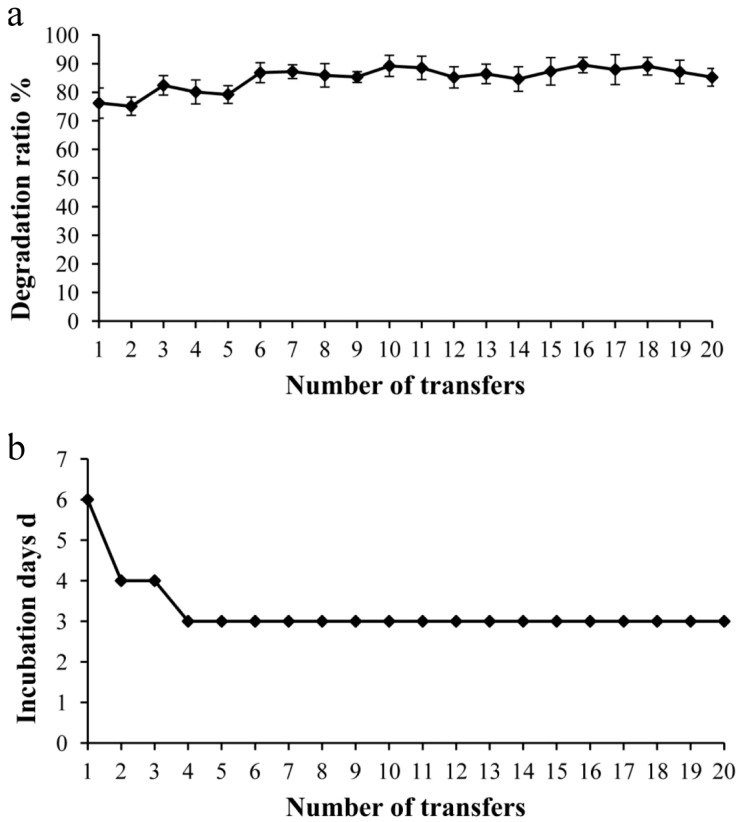
(**a**) Filter paper and rice straw degradation ratio (%) during the enrichment process; (**b**) Days of transfers during the enrichment process.

For the enrichment of cellulolytic consortium, hindgut samples of *H. parallela* larvae were collected. A filter paper strip was used as an indicator of cellulase activity. After 20 subcultures, the consortium still showed stable cellulolytic activity, and the filter paper was mostly decomposed after incubation for three days (Figure S2), which indicated its high efficiency for cellulose degradation. The culture digested over 85% of the total rice straw and filter paper strip on average within three days, and the relative standard deviations of the degradation ratio during the 10th to 20th subcultures were about 2.0%, suggesting the weight losses remained stable (Figure 1).

### 2.2. Bacterial Communities in the Different Groups

Time-course dynamics of the microbial structure of the consortium in the subcultivation procedure were analyzed using the samples from the 0th (T0), 10th (T10), and 20th (T20) subcultivations. Bacterial diversities and compositions of these three samples were investigated using high-throughput 16S rRNA gene-based pyrosequencing method. From our results, we found that the rarefaction curves showed a clear saturation, indicating that the bacterial community was well represented in this study (Figure S3).

**Figure 2:**
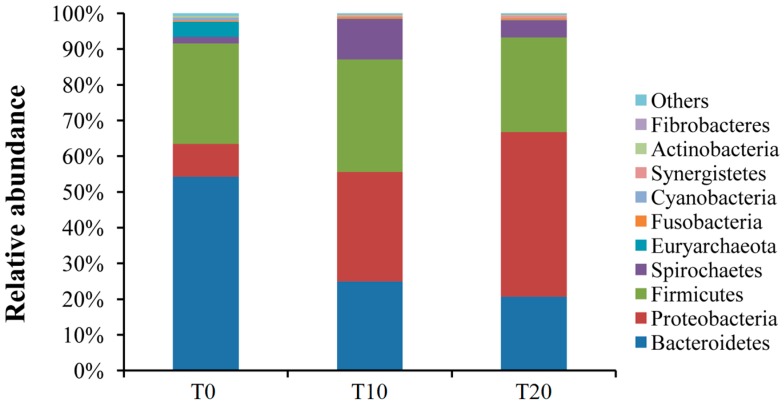
Bacterial composition of the communities in those three groups (Phylum level). Note: T0, T10, and T20 means that the number of transfers in each was 0, 10, and 20, respectively.

At the phylum level, for these three samples, around 99% of the sequences could be classified. The top nine phyla included Bacteroidetes, Proteobacteria, Firmicutes, Spirochaetae, Euryachaeota, Fusobacteria, Cyanobacteria, Synergistetes, Actinobacteria, and Fibrobacteres. The majority of bacterial sequences in these three groups belonged to these phyla Bacteroidetes, Proteobacteria, and Firmicutes, which represented 54.32%, 9.13%, and 28.08% of each of the total sequences for the T0 group; 24.93%, 30.69%, and 31.47% of each of the total sequences for the T10 group; and 20.73%, 46.05%, and 26.46% of each of the total sequences for the T20 group, respectively (Figure 2, Table S1).

**Figure 3:**
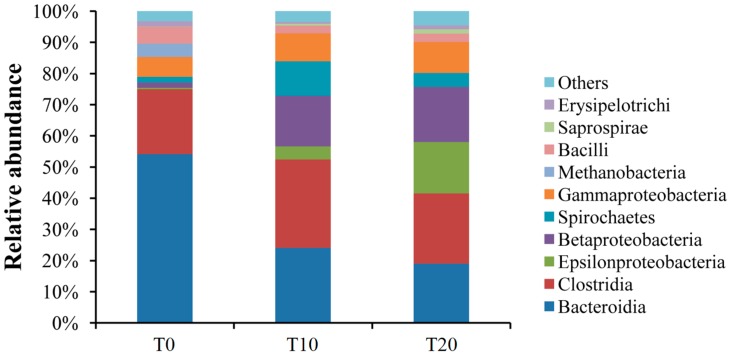
Bacterial composition of the communities in those three groups (Class level). Note: T0, T10, and T20 means that the number of transfers in each was 0, 10, and 20, respectively.

When sequences were analyzed at the class level, around 96% of the sequences could be classified; the bacterial taxa were distributed in Bacteroidia, Clostridia, Epsilonproteobacteria, Betaproteobacteria, Spirochaetes, Gammaproteobacteria, Methanobacteria, Bacilli, Saprospirae, and Erysipelotrichi. Bacteroidia was the dominant bacterial class in the T0 group (54.14%), followed by Clostridia (20.87%) and Gammaproteobacteria (6.42%). For the T10 group, Clostridia was the dominant bacterial class (28.38%), followed by Bacteroidia (24.05%), Betaproteobacteria (16.22%), and Spirochaetes (11.09%). For the T20 group, Clostridia was also the dominant bacterial class (22.52%), followed by Bacteroidia (19.02%), Betaproteobacteria (17.73%), and Epsilonproteobacteria (16.48%) (Figure 3, Table S2).

**Figure 4:**
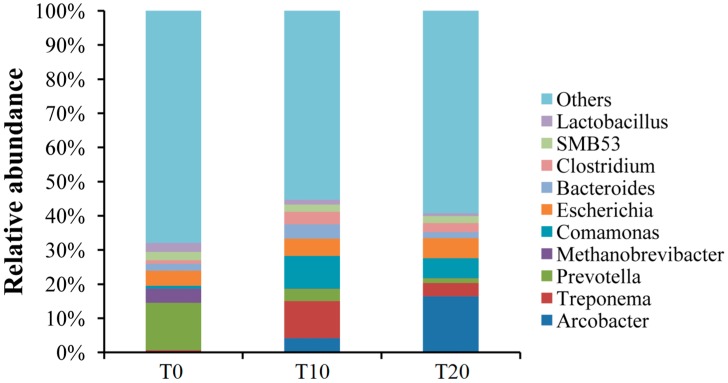
Bacterial composition of the communities in those three groups (Genus level). Note: T0, T10, and T20 means that the number of transfers in each was 0, 10, and 20, respectively.

When sequences were analyzed at the genus level (the lowest level assigned), only around 32%–45% of the sequences could be classified. For the T0 group, 13.85% of the sequences belonged to the genus *Prevotella*, followed by more than 4% of the genera *Escherichia* (4.38%) and *Methanobrevibacter* (4.20%). For the T10 group, *Treponema* (10.82%) was the most dominant bacterial genus, followed by more than 4% of the genera *Comamonas* (9.51%), *Escherichia* (5.09%), *Arcobacter* (4.20%), and *Bacteroides* (4.20%). For the T20 group, *Arconacter* (16.47%) was the most dominant bacterial genus, followed by more than 4% of the genera *Comamonas* (5.83%) and *Escherichia* (5.82%) (Figure 4, Table S3).

### 2.3. Dynamics of the Microbial Community Structure

**Figure 5:**
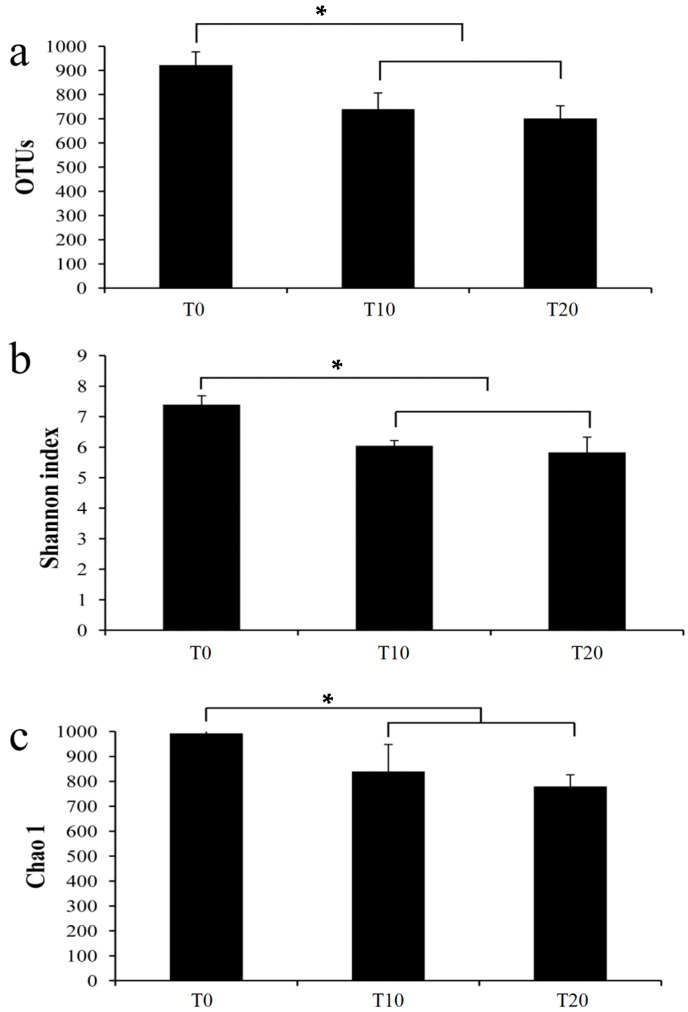
Alpha diversities of those three groups. Note: T0, T10, and T20 means that the number of transfers in each was 0, 10, and 20, respectively. * means that there were significant differences between the T0 and T10 or T20 groups at the level of 0.05. There were no significant differences between the T10 and T20 groups. (**a**) The OTU numbers of all samples; (**b**) The Shannon index of all samples; and (**c**) The Chao1 index of all samples.

An average of 40,846, 37,991 and 36,749 effective tags were obtained from the T0, T10, and T20 groups, respectively, and all further analyses were performed on these effective tags. At a >97% sequence identity threshold, the T0 group (922) showed a significantly higher OTUs than those of the T10 (739) and T20 (701) groups (Figure 5a). The Shannon diversity index, evaluated at 97% similarity, showed a similar comparative trend in predicting the number of OTUs. Samples from the T0 group (7.40) showed the highest value, followed by the T10 (6.04) and T20 (5.83) groups (Figure 5b). For the richness index of Chao1, we also found that the T0 group (992.51) showed the highest value, followed by the T10 group (839.41); the T20 group showed the lowest value (779.22) (Figure 5c). For these three indexes, the T0 group showed significantly higher values than those of the other two groups; however, there were no significantly differences between the T10 and T20 groups. These results indicated that the bacterial diversity and richness decreased during the consortium enrichment process.

**Figure 6:**
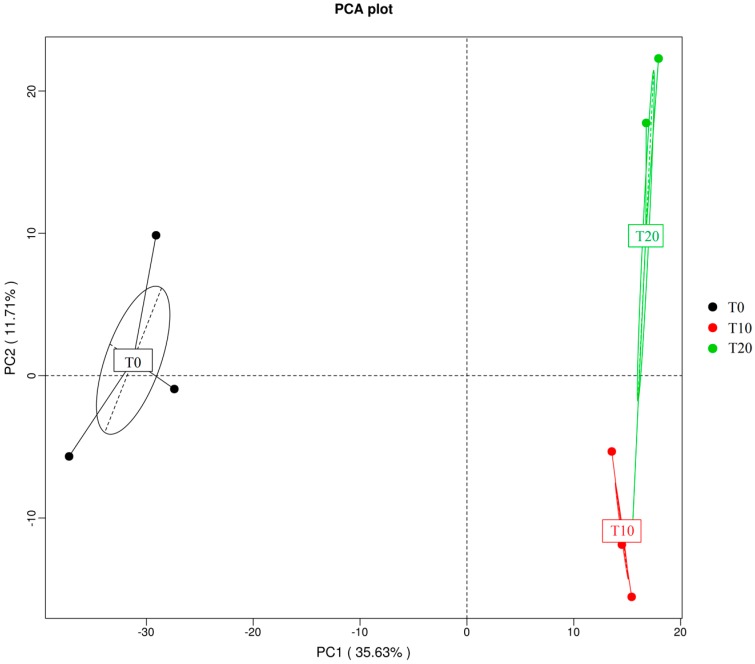
Principal component analysis (PCA) of the bacterial communities of those three groups. Note: T0, T10, and T20 means that the number of transfers in each was 0, 10, and 20, respectively. These ovals represent the error ellipse, which means error distribution in each direction of each samples.

In the present study, the comparison of bacterial communities by principal component analysis (PCA) showed that the bacterial communities of these three groups were separated from each other (Figure 6), indicating a distinct shift in bacterial community composition during the consortium enrichment process.

At the phylum level, we found that Bacteroidetes abundance in group T0 was significantly higher than those of the T10 and T20 groups (*p* < 0.05); however, the abundance of Proteobacteria obviously increased during the consortium enrichment process (*p* < 0.05, Table S1). For Spirochaetes, it was increased from the 0th (T0) to the 10th (T10) subcultivations and then decreased (Table S1).

At the class level, the abundance of Bacteroidia in the T0 group was significantly higher than those of the T10 and T20 groups (*p* < 0.05). However, Betaproteobacteria, Clostridia, and Spirochaetes were significantly less abundant in the T0 group (*p* < 0.05, Table S2). In addition, we also found that there was no significant difference in the top 10 bacterial classes between the T10 and T20 groups.

At the genus level, we found that the abundance of *Arcobacter* and *Clostridium* markedly increased during the consortium enrichment process (*p* < 0.05). However, *Prevotella* was sharply decreased during the consortium enrichment process (*p* < 0.05). *Treponema* and *Comamonas* were increased from the 0th (T0) to the 10th (T10) subcultivations and then decreased (Table S3).

### 2.4. Lignocellulosic Materials Degradation

**Figure 7:**
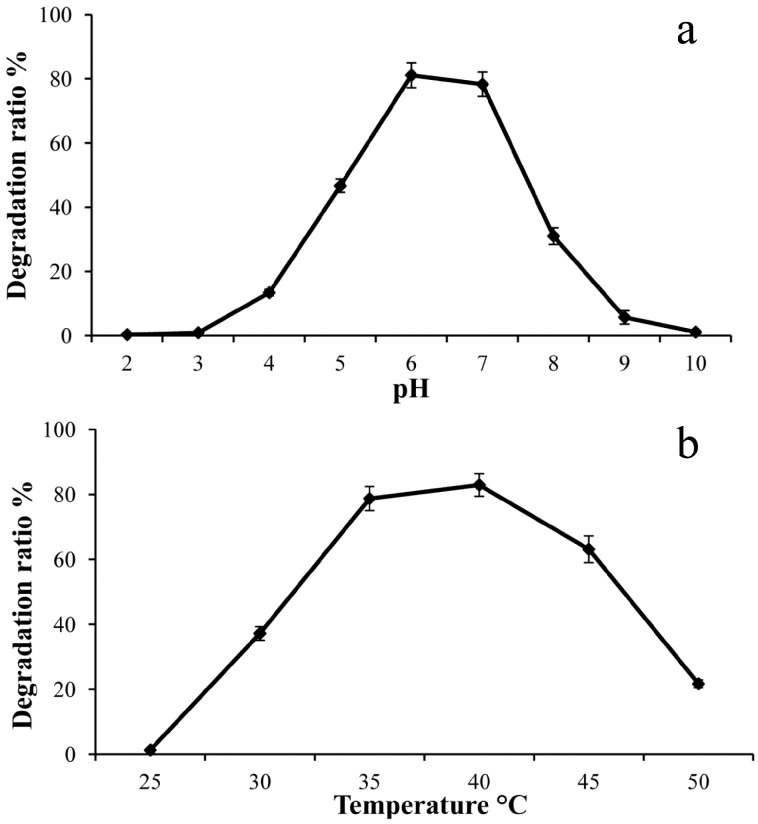
Effect of (**a**) pH and (**b**) temperature on the degradation ratio of rice straw.

Rice straw, as a main agro-industrial residue, has great potential to be converted into energy in order to meet the countries’ (India, China, etc.) energy demands, and it has by now received a great deal of attention. In this study, the degradation ratio of rice straw after three days of cultivation was determined. The degradation ratio of rice straw was detected over broad pH ranges (2.0 to 10.0); the maximum degradation ratio was observed at pH 6.0 (81.1%) (Figure 7a). Figure 7b shows the temperature profile for the degradation. The highest degradation ratio (82.9%) was observed at 40 °C. Under the optimal conditions, the degradation ratio for rice straw was about 83.1%.

## 3. Discussion

In recent years, rice straw has received a lot of attention as an important source of renewable energy. Many rice-producing countries can enjoy the environmental and economic benefits of the utilisation of rice straw, and the biological pretreatment of rice straw is at present the most attractive alternative due to environmental concerns [15]. Until now, several cellulolytic consortia have been enriched from different ecosystems, such as soils [3], composts [4,8], and so on. However, the phytophagous insects have not yet received enough attention. Throughout the course of evolution, these insects have established symbiotic interactions with different microorganisms that perform cellulolytic and xylanolytic activities and thus are highly efficient natural bioreactors [8,14,16]. In this study, hindgut samples were collected from the *H. parallela* larvae, and a stable cellulolytic consortium was obtained. The bacterial consortium showed high efficiency for rice straw degradation with short incubation time (indicating its strong cellulolytic activity), and could be considered a potential candidate for use in commercial biomass conversion. We also found that the degradability of rice straw was lower than filter paper, and similar results were also found in some previous studies [3,17]. It is well known that, due to the presence of lignin in the native lignocelluloses, they are more resistant than pure cellulose to microbial degradation [3].

The enrichment process is the selective adjustment of the composition and structure of the microbial community, and only the microorganisms that can adapt to the environment can survive [3]. Similar results were found in this study; the bacterial diversity and richness decreased during the consortium enrichment process, suggesting that convergent adaptation is driven by the selective pressure applied during the enrichment process. Furthermore, we also found that both aerobic and anaerobic bacteria coexisted steadily in the enriched consortium. This feature would be caused by the culture conditions; the upper phase of the culture system would supply oxygen for the aerobic bacteria, and the lower phase would be in anaerobic conditions [18].

Microbial structure analysis showed that the lignocellulose, such as rice straw and filter paper, led to significant enrichment of the phyla Proteobacteria and Spirochaetes, classes Clostridia, Epsilonproteobacteria, and Betaproteobacteria, and genera *Arcobacter*, *Treponema*, *Comamonas*, and *Clostridium*. Consulting the collection of cellulolytic enzyme sequences in the CAZy database, we found that the Proteobacteria phylum predominated, and our previous studies also showed that Proteobacteria is the most abundant bacterial phylum in the hindgut of *H. parallela* larvae. Many cellulolytic enzyme sequences were also closely related to enzymes of this phylum [13]. Spirochetes is the dominant phylum in the higher termite species, and metagenomic analysis has revealed that it is responsible for cellulose and hemicellulose utilization in the termite. Furthermore, another metagenomic study on cow rumen also revealed that the bacteria of the phylum Spirochaetes were always adherent to and degraded the plant fiber materials [19]. These results indicated that these two phyla might have strong cellulolytic activities in the enrichment consortium. For the genus *Treponema* of the phylum Spirochetes, previous studies also showed that this genus was the dominant anaerobic bacterial genus in the wood-feeding termite, and they encoded glycoside hydrolase gene modules [20]. For *Comamonas*, previous studies have shown that different *Comamonas* species of the class Betaproteobacteria have previously been reported in termites and eroded bamboo slips, and are known to be involved in lignin, cellulose, and hemicellulose degradation [21,22]. In addition, sequences related to those of some Clostridia are known to produce various plant biomass-degrading enzymes, including cellulase and hemicellulase [23,24]. The genus *Clostridium* of the class Clostridia are well known as a typical anaerobic cellulolytic genus; they are reported as the important plant biomass degraders in anoxic conditions, and they usually have a cellulosome, which is a cellulolytic enzyme complex well known in anaerobic cellulose-degrading bacteria and able to effectively degrade lignocellulosic materials into acids, alcohols, biogas, and hydrogen effectively [25,26]. These results suggest that those bacteria might be important members of cellulolytic microorganisms in our enriched bacterial consortium. However, since only about half of the sequences could be identified at the genus level, we still need further study to elucidate the accurate function of these bacteria in the enrichment consortium; such study may include a combination of pure culture techniques, metagenomics, and metatranscriptome analysis.

Aside from the potential cellulolytic bacteria from the genera *Treponema*, *Comamonas*, and *Clostridium*, the most interesting finding of this study is that significant numbers (16.47% of 40.71% of classified sequences) of bacteria related to the genus *Arcobacter,* which prefer aerobic and microaerobic conditions and are potentially pathogenic representatives, were present in the 20th subcultivation sample. A similar result was also found in an activated sludge sample, and the *Arcobacter* might be essentially a bioreactor with biomass recycling in this ecosystem [27]. Previous studies have shown that the genus *Arcobacter* has been frequently isolated from a wide range of environments, and is strongly associated with nitrogen-fixing and sulfide-oxidizing activities [28,29]. Some previous studies also showed that the coexistence of anaerobic cellulolytic and aerobic non-cellulolytic bacteria (which scavenge metabolites from cellulose) is crucial for cellulose degradation, and this phenomenon is often detected at various sites when cellulose degradation occurs [18,30]. The aerobic bacteria would consume oxygen by utilizing substrates contained in peptone and yeast extract, and would supply the anaerobic environment, reduce the concentration of cellooligosaccharides, and neutralize the pH value for the anaerobic bacteria, which would accelerate the cellulose degradation process [18]. Although the high-throughput 16S rRNA gene-based pyrosequencing method revealed this surprising finding, the accurate function of the genus *Arcobacter* would require further study.

In recent years, many lignocellulose-degrading thermophilic consortia have been constructed [4,8], but mesophilic cellulolytic consortia would also be desirable, and they could offer advantages in some biological hydrolysis of cellulose. For example, the biological generation of hydrogen from fermentation of renewable resources, including biomass-provide economic and environmental benefits, and low pH (5.5–6.0) and temperature were optimal for biological hydrogen production by repressing the activities of hydrogen consumers [31,32]. Butanol, as an aliphatic saturated alcohol, from fermentation of biomass, can be used as a solvent for a wide variety of chemical and textile industry applications. Normally, it is also produced by mesophilic bacteria [33]. In this study, we found that the consortium showed the maximum degradation ratio at moderate conditions, indicating that it might offer advantages in bio-hydrogen and butanol production. However, this still needs further product analysis during rice straw degradation.

## 4. Materials and Methods

### 4.1. Ethics Statement

The peanut field is private property, and we have obtained prior permission to enter the peanut field to catch the larvae. The larvae are safe, and none of the laws and rules of the Government of China prohibit their research.

### 4.2. Insect Samples

Healthy early third-instar larvae were collected from a wild field (Jingmen, Hubei, China). They were kept in a plastic container, which was filled with soil. These larvae reared under a temperature of 27 ± 1 °C and a light-dark photoperiod of 14:10 h [13]. Larvae were fed with peanut roots until they were used. Insect dissections were performed according to the method described by Zhang and Jackon [10]. First, these larvae were washed twice with 75% ethanol, and then rinsed twice in sterile distilled water. Eighty entire hindguts of larvae were removed under sterile conditions. They were homogenized for further analyses.

### 4.3. Consortium Enrichment

For this experiment, rice straw was used as the cellulosic material. Rice straw produced in Jiangxi was dried. It was cut into pieces and soaked in a solution of 15 g/L NaOH for 48 h, washed to a neutral pH by flowing water, and then dried in an oven at 105 °C. These dried rice straw were ground in a Wiley mill with a 2-mm screen.

The cellulolytic microbial consortium was obtained and prepared as described by Wongwilaiwalin et al. [4] with some modification. Five grams of homogenized hindgut samples were transferred into 50 mL of water and shaken for 1 h, then 5 mL of the suspension was inoculated into 100 mL of an autoclaved peptone cellulose solution (PCS) medium (0.5% peptone (Solarbio, Beijing, China), 0.1% yeast extract (Solarbio), 0.15% CaCO_3_ (Solarbio), 0.5% NaCl (Solarbio), pH 7.0) supplemented with 1% alkali-pretreated rice straw, and with a filter paper strip (0.3 g, 1 × 6 cm, Whatman) as an indicator of cellulase activity [4]. The mixture was incubated at 37 °C under static conditions. When the filter paper strip was almost decomposed, 5 mL of culture was transferred into a fresh PCS medium with rice straw and filter paper strip, as described above (Figure S1). The remaining culture was filtered. The solid filtration was then suspended in 100 mL of acetic-nitric reagent and heated at 100 °C for 30 min to remove the biological cells. Then, the acetic-nitric treated suspension was filtered again. The residual cellulose was washed three times with 100 mL of distilled water each time. After washing and filtration, the filtered solids were dried at 80 °C and weighed [3]. The degradation ratio of rice straw and filter paper was calculated as per the following formula, and it was used for investigation of the degradation capacity of the enriched microbial consortium:

Degradation ratio% = (1 − dry residual cellulose weight/1.3) × 100%

where 1.3 is the total weight of the rice straw and filter paper strip before degradation.

This procedure was repeated several times, and the consortium was not obtained until the degradation ratio was kept stable (relative standard deviations <0.05) for at least ten transfers [3].

### 4.4. DNA Extraction, 16S rRNA Amplification from the Microbial Consortium

Total genomic DNA from the original (0th), 10th, and 20th subcultivations was extracted using a MoBio UltraClean™Soil DNA isolation kit (SanDiego, CA, USA) following the manufacturer’s instructions. Finally, the DNA was eluted with TE buffer (Tris-EDTA buffer). DNA concentration and purity were measured using the NanoDrop ND-1000 (NanoDrop Technologies, Wilmington, DE, USA). The total DNA was stored at −80 °C before use.

The bacterial 16S rRNA variable V4 region was used to identify bacterial composition. Two universal primers (515F and 806R), containing the specific barcode sequence, were used for the amplification of the V4 region in this study (515F: 5’-GTTTCGGTGCCAGCMGCCGCGGTAA-3’, 806R: 5’-GTGAAAGGACTACHVGGGTWTCTAAT-3’), where the barcode sequence is shown in italics. The PCR reaction was performed in 30 μL with 15 μL of Phusion^®^ High-Fidelity PCR Master Mix (New England Biolabs, Ipswich, MA, USA), 0.2 μM of each primer, and approximately 10 ng of template DNA. The amplification procedure was as follows: 98 °C for 1 min, followed by 30 cycles of denaturation at 98 °C for 10 s, annealing at 50 °C for 30 s, and elongation at 72 °C for 60 s. Finally, extension occurred for 10 min at 72 °C.

### 4.5. PCR Product Quantification, Qualification, Purification, and Sequencing

A 1× loading buffer was mixed (containing SYBR green) with PCR products, and electrophoresis was run on 2% agarose gel for detection. PCR products were mixed with equal portions. Then, the mixture of PCR products was purified with a GeneJET Gel Extraction Kit (Thermo Scientific, Schwerte, Germany).

Sequencing libraries were constructed using the NEBNext DNA sample preparation kit following the manufacturer’s recommendations. The library concentration and quality were assessed on the Qubit@ 2.0 Fluorometer (Thermo Scientific) and Bioanalyzer 2100 (Agilent, Palo Alto, CA, USA) system. The sequence libraries were analyzed using an Illumina MiSeq platform.

### 4.6. Degradation of Rice Straw

The microbial consortium enriched as above was used to determine its cellulolytic activity. The culture was inoculated into the PCS medium supplemented with 1% rice straw as cellulosic substrate. The effect of pH and temperature on the degradation of rice straw was studied by varying the incubation medium pH (2.0, 3.0, 4.0, 5.0, 6.0, 7.0, 8.0, 9.0, and 10.0) and the incubation temperature (20, 25, 30, 35, 40, 45, and 50 °C). The incubation was cultured for 3 days under static conditions. The degradation ratio of rice straw was calculated as described above.

### 4.7. Data Analysis

Paired-end reads were assembled using FLASH [34]. The effective sequences, with ≥97% similarity, were then assigned into operational taxonomic units (OTU) using the UPARSE-OTU and UPARSE-OTUref algorithms of UPARSE software package (Uparse v7.0.1001) [35]. Alpha (within samples, including three metrics: Chao1, Observed Species, and the Shannon index) and beta (among samples) diversity were analyzed using an in-house developed Perl scripts. These three metrics were used to generate the rarefaction curves. Then, representative sequences for each OTU were picked, and the taxonomic information for each sequence was analyzed by the RDP classifier (Version 2.2) [36]. Principal component analysis (PCA) was performed using the multivariate statistical software package PC-ORD v. 6.12 [37]. Graphical displays were visualized using a Krona chart [38].

### 4.8. Statistical Analysis

All values in this study were averaged from the three experiments. The data were analyzed using a one-way ANOVA and Duncan’s multiple comparison test (*p* < 0.05) (SPSS 16.0 software, SPSS, Inc., Chicago, IL, USA).

## 5. Conclusions

In this study, the microbial consortium degraded about 83.1% of rice straw within three days, indicating strong cellulolytic activity. The bacterial diversity and richness decreased during the enrichment process, and bacterial structure analysis showed that the consortium enrichment process (supplied with lignocellulose materials) can lead to a significant enrichment in phyla Proteobacteria and Spirochaetes, classes Clostridia, Epsilonproteobacteria, and Betaproteobacteria, and genera *Arcobacter*, *Treponema*, *Comamonas*, and *Clostridium—*some of which are well-known as cellulolytic microorganisms. However, further studies are still needed to isolate the key bacteria from the enriched consortium and to study the function and synergistic reaction of these microbes.

## Supplementary Materials

Supplementary materials can be found at www.mdpi.com/1422-0067/17/10/1646/s1.
