# Supplementary material for: Construction and Characterization of a Cellulolytic Consortium Enriched from the Hindgut of Holotrichia parallela Larvae
Source: Int J Mol Sci. 2016 Sep 30;17(10):1646. doi: 10.3390/ijms17101646 (PMC5085679; doi:10.3390/ijms17101646)
Supplement: Supplementary file 1 [file ijms-17-01646-s001.pdf]

Supplementary Materials: Construction and Characterization of a Cellulolytic Consortium Enriched from the Hindgut of *Holotrichia parallela* Larvae

Ping Sheng, Jiangli Huang, Zhihong Zhang, Dongsheng Wang, Xiaojuan Tian and Jiannan Ding

Table S1. Bacterial compositions and comparative analysis of these bacteria (phylum level).

Taxa	Relative Abundance %(OM Group)	Relative Abundance %(MM Group)	Relative Abundance %(FM Group)	*p* Value (OM vs. MM)	*p* Value (MM vs. FM)	*p* Value (OM vs. FM)
Bacteroidetes	54.32	24.93	20.73	0.006	0.130	0.005
Proteobacteria	9.13	30.68	46.05	0.012	0.013	0.005
Spirochaetes	1.85	11.32	4.77	0.036	0.078	0.083

Table S2. Bacterial compositions and comparative analysis of these bacteria (class level).

Taxa	Relative Abundance %(OM Group)	Relative Abundance %(MM Group)	Relative Abundance %(FM Group)	*p* Value (OM vs. MM)	*p* Value (MM vs. FM)	*p* Value (OM vs. FM)
Bacteroidia	54.14	24.05	19.02	0.005	0.134	0.001
Clostridia	20.87	28.38	22.52	0.036	0.102	0.718
Betaproteobacteria	1.69	16.22	17.73	0.001	0.636	0.023
Epsilonproteobacteria	0.40	4.21	16.48	0.026	0.052	0.011
Spirochaetes	1.83	11.09	4.42	0.041	0.067	0.118
Bacilli	5.45	2.48	2.69	0.048	0.869	0.019

Table S3. Bacterial compositions and comparative analysis of these bacteria (genus level).

Taxa	Relative Abundance %(OM Group)	Relative Abundance %(MM Group)	Relative Abundance %(FM Group)	*p* Value (OM vs. MM)	*p* Value (MM vs. FM)	*p* Value (OM vs. FM)
Prevotella	13.85	3.65	1.40	0.006	0.001	0.003
Comamonas	0.82	9.51	5.83	0.005	0.153	0.041
Arcobacter	0.40	4.20	16.47	0.015	0.064	0.033
Treponema	0.29	10.82	3.89	0.026	0.084	0.040
Clostridium	1.01	3.66	2.69	0.049	0.478	0.013
Lactobacillus	2.66	1.38	0.81	0.147	0.582	0.007

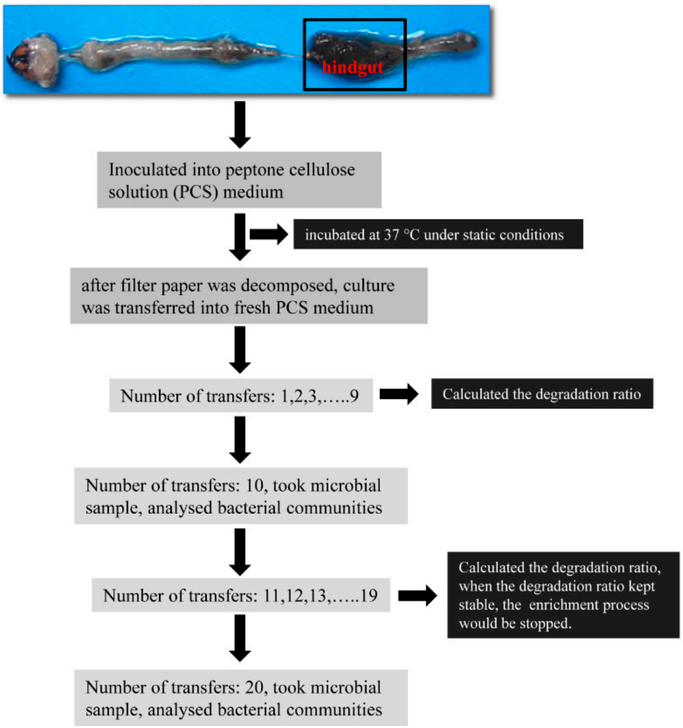

Figure S1. Image of the enrichment method.

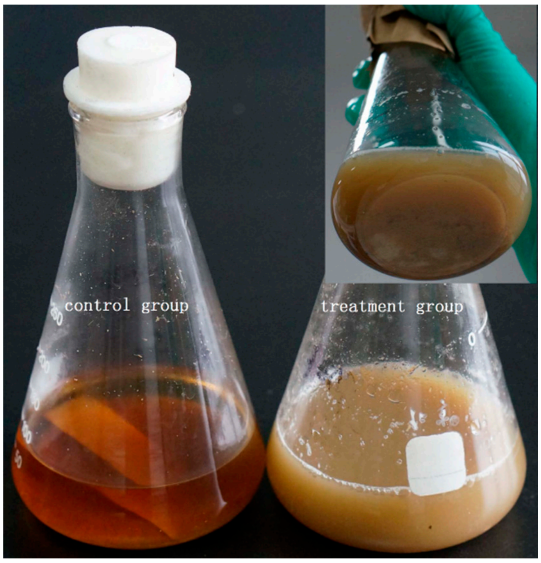

Figure S2. Picture of decomposed filter paper after 3 days of cultivation.

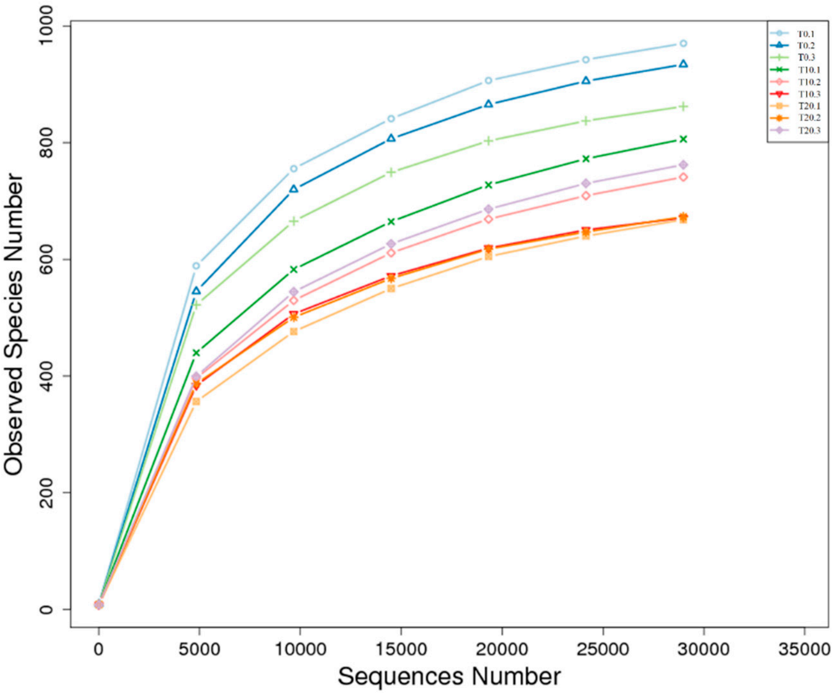

Figure S3. Rarefaction curves of all samples.
